# Supplementary figures and images for: Robust Normalization of Luciferase Reporter Data
Source: Methods Protoc. 2019 Jul 25;2(3):62. doi: 10.3390/mps2030062 (PMC6789503; doi:10.3390/mps2030062)

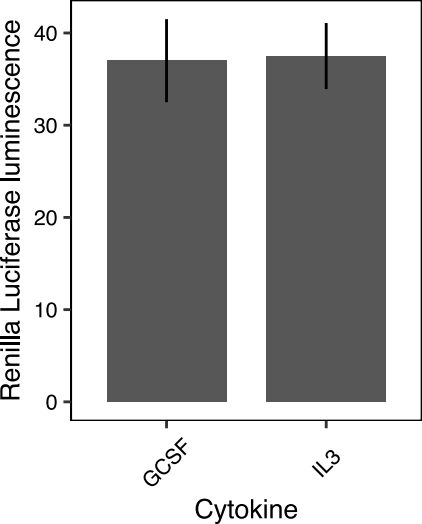

Supplement: Supplementary file 1 [file mps-02-00062-s001.zip › mps-539565-S1.pdf]
